# Pyridoxine (Vitamin B_6_) and the Glutathione Peroxidase System; a Link between One-Carbon Metabolism and Antioxidation

**DOI:** 10.3390/nu9030189

**Published:** 2017-02-24

**Authors:** Danyel Bueno Dalto, Jean-Jacques Matte

**Affiliations:** 1 Sherbrooke Research Centre, Agriculture and Agri-Food Canada, Sherbrooke, QC J1M 0C8, Canada; danyel.buenodalto@agr.gc.ca; 2 Department of Biology, Université de Sherbrooke, Sherbrooke, QC J1K 2R1, Canada

**Keywords:** glutathione peroxidase, one-carbon, pig, pyridoxine, remethylation, transsulfuration

## Abstract

Vitamin B_6_ (B_6_) has a central role in the metabolism of amino acids, which includes important interactions with endogenous redox reactions through its effects on the glutathione peroxidase (GPX) system. In fact, B_6_-dependent enzymes catalyse most reactions of the transsulfuration pathway, driving homocysteine to cysteine and further into GPX proteins. Considering that mammals metabolize sulfur- and seleno-amino acids similarly, B_6_ plays an important role in the fate of sulfur-homocysteine and its seleno counterpart between transsulfuration and one-carbon metabolism, especially under oxidative stress conditions. This is particularly important in reproduction because ovarian metabolism may generate an excess of reactive oxygen species (ROS) during the peri-estrus period, which may impair ovulatory functions and early embryo development. Later in gestation, placentation raises embryo oxygen tension and may induce a higher expression of ROS markers and eventually embryo losses. Interestingly, the metabolic accumulation of ROS up-regulates the flow of one-carbon units to transsulfuration and down-regulates remethylation. However, in embryos, the transsulfuration pathway is not functional, making the understanding of the interplay between these two pathways particularly crucial. In this review, the importance of the maternal metabolic status of B_6_ for the flow of one-carbon units towards both maternal and embryonic GPX systems is discussed. Additionally, B_6_ effects on GPX activity and gene expression in dams, as well as embryo development, are presented in a pig model under different oxidative stress conditions.

## 1. Introduction

Among various metabolic pathways present in the organism, the metabolism of sulfur- (S) methionine and its seleno- (Se) analogous (Se-methionine) are particularly important because they not only contribute to protein mass but also produce (Se) homocysteine, a key metabolite connecting two fundamental metabolic functions, the one-carbon metabolism and the antioxidative system.

The transfer of one-carbon groups, represented by methyl (–CH3), methylene (–CH2–), formyl (–CHO), formimino (–CHNH), and methenyl (–CH=), is involved in the remethylation of (Se) homocysteine to (Se) methionine, as well as related pathways such as the folate cycle and the choline oxidation pathway [1]. Additionally, during the demethylation of (Se) methionine to (Se) homocysteine, the universal bioactive methyl donor S-adenosylmethionine (SAM) is synthesized and donates its methyl group to a large number of methyl acceptors catalyzed by methyltransferases [1,2] with profound impacts on DNA synthesis, protection and repair, cellular metabolism, and cell proliferation and, consequently, with direct effects on embryo/fetal growth [3,4].

During redox challenges, however, the high level of reactive oxygen species (ROS) induces a negative feedback on methionine synthase (MTR) [5] and betaine-homocysteine S-methyltransferase (BHMT) [6], which are enzymes that catalyze the regeneration of methionine from homocysteine [7]. Concomitantly, a positive feedback on cystathionine β-synthase (CBS), the first enzyme of the transsulfuration pathway, increases the flow of carbon towards transsulfuration reactions [8].

**Figure 1:**
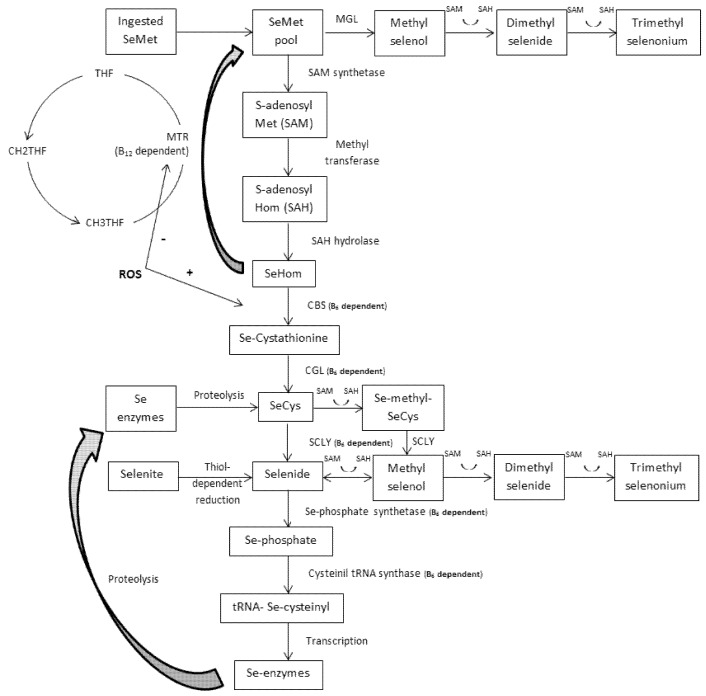
Transmethylation and transsulfuration pathways of selenium (similar for sulfur counterparts) in adults. Selenomethionine (SeMet) is methylated to methyl selenol by methionine γ lyase (MGL) or demethylated to selenohomocysteine (SeHom). SeHom may follow remethylation back to SeMet or transsulfuration to form selenocysteine (SeCys). The substrate-specific enzyme selenocysteine lyase (SCLY), which represents the landmark between sulfur and selenium metabolisms, catalyses the synthesis of selenide from SeCys. Selenide may also be formed from dietary selenite after thiol-dependent reactions. Selenide may be methylated and excreted or phosphorylated and its selenium (Se) incorporated into tRNA Se-cysteinyl with further translation to Se-enzymes. THF = tetrahydrofolate; CH2THF = 5,10-methylene-THF; CH3THF = 5-methyl-THF; MTR = methionine synthase; CBS = cystathionine β synthase; CGL = cystathionine γ lyase; ROS = reactive oxygen species.

The transsulfuration of homocysteine, catalyzed by two vitamin B_6_- (B_6_) dependent reactions, consumes one-carbon units and is a major source of cysteine, contributing with about 50% of the cysteine used for glutathione (GSH) synthesis [8], the major redox buffer in mammalian cells. Se-homocysteine follows these same B_6_-dependent steps of the transsulfuration pathway but syntheses Se-cysteine (SeCys). Due to the action of the B_6_-dependent and Se-specific enzyme selenocysteine lyase (SCLY), SeCys loses its organic part and releases selenide. Selenide is also formed after dietary selenite (mineral Se) supplementation [9]. Considering the high cytotoxicity of selenide, it has to be immediately metabolized through either methylation reactions consuming SAM or phosphorylation to merge Se into an organic moiety in a B_6_-dependent reaction (Figure 1). After another B_6_-dependent reaction, phosphorylated Se is incorporated into tRNA Se-cysteinyl with further translation into Se-proteins such as Se-dependent glutathione peroxidases (SeGPX), the enzyme responsible for the oxidation of GSH during the important detoxification of ROS [10].

Although the transmethylation of (Se) methionine is not dependent on B_6_ [11], the direct impact of this vitamin on the transsulfuration of (Se) homocysteine to (Se) cysteine and the intrinsic relation between these two metabolic pathways make the B_6_ status of an individual important not only for the antioxidant system but for the one-carbon pool as well. Considering that the transsulfuration of homocysteine to the synthesis of GSH requires two B_6_-dependent reactions, whereas Se-homocysteine requires five B_6_-dependent reactions to synthesize SeGPX, the impact of B_6_ is expected to be more evident for Se metabolism. Additionally, the dissimilar metabolisms between organic and mineral Se, including different mechanisms of regulation for the synthesis of selenoprotein, indicate possible different fates for the one-carbon metabolism. 

All the above concepts are even more fundamental in developing embryos because of the singular transfer of Se to pre-implantation embryos [12], the redox alterations brought by the placentation process [13], and the incomplete transsulfuration pathway [14]. 

The present review discusses the impact of B_6_ on the equilibrium between the synthesis and the consumption of one-carbon units under different oxidative stress conditions, focusing not only sulfur-related metabolism but also Se metabolism and the differences between Se sources both in adults and embryos at 5- and 30-days of gestation using a pig model.

## 2. Vitamin B_6_ Metabolism

### Vitamers and Their Metabolism

Vitamin B_6_ is a general description of six interconvertible metabolites, pyridoxine (alcohol), pyridoxamine (amine), pyridoxal (aldehyde), and their respective phosphates. Its main metabolic form differs between plant (pyridoxine) and animal (pyridoxal and/or pyridoxamine) sources, but, independently of origin, they are mainly found in the phosphorylated state or bound to proteins [15]. 

The enteric absorption of the phosphorylated protein-bound B_6_ vitamers, mostly at jejunum and ileum, is dependent on their dephosphorylation by membrane-bound alkaline phosphatases present in the intestinal mucosa [16]. All three dephosphorylated vitamers are absorbed by passive diffusion, and, within the enterocytes, these metabolites are re-phosphorylated, generating a metabolic trapping of the vitamin with further oxidation to pyridoxal-5-phosphate (P-5-P), its metabolically active form [17]. This latter metabolite must be dephosphorylated at the intestinal serosal surface before its release into the portal circulation. Dephosphorylated forms of B_6_ are readily taken up by membranes, whereas the phosphorylated analogs are not; therefore, phosphorylation may be considered a mechanism for the intracellular retention of this vitamin [15].

Pyridoxal released into the portal circulation is absorbed in the liver by passive diffusion, followed by re-phosphorylation within the cells. In order to cross liver cell membranes, P-5-P is hydrolyzed to pyridoxal and released into the general circulation bound to albumin and/or hemoglobin [18]. Any P-5-P that is not bound to proteins is readily hydrolyzed and the free pyridoxal remaining in the liver is oxidized to 4-pyridoxic acid and excreted. 

Considering that no specific tissular storage of B_6_ is present in the organism, both short- and long-term whole body pools of B_6_ (about 12 h and 1 month, respectively) are present as P-5-P bound to enzymes/proteins [15]. Blood plasma is the major source of extrahepatic B_6_, which occurs mainly as P-5-P bound to albumin [16]. Circulatory P-5-P must be hydrolyzed (by extracellular alkaline phosphatases) to pyridoxal that can cross cell membranes and then is trapped intracellularly by phosphorylation. Pyridoxal-5-P is over six times more concentrated in erythrocytes than in plasma possibly because the Schiff base with hemoglobin is stronger than the one with albumin, driving the uptake of the vitamin to erythrocytes; however, its storage in these cells is saturable [16,19].

## 3. Vitamin B_6_ Metabolic Functions

In addition to the role of B_6_ as a cofactor for the degradation of stored carbohydrates [20] and its function as a protective agent against ROS generated in vitro [21], P-5-P was shown to interact with steroid hormone-receptors, affecting their genes expression [22]. Pyridoxal-5-P also acts as a cofactor in the biosynthesis of many neurotransmitters such as dopamine, epinephrine, gamma-aminobutyric acid, histamine, norepinephrine, and serotonin, as well as the neuromodulator serine [17]. However, the most recognized role of P-5-P is the catalysis of many important steps in the metabolism of amino acids, such as transamination, racemization, decarboxylation, and α,β-elimination reactions [23,24]. The various reactions of P-5-P in the metabolism of amino acids depend on its ability to stabilize amino acid carbanions. In the absence of a substrate, P-5-P forms an internal aldimine (Schiff base) with the lysyl residue (ε-amino group) of the enzyme. Once a substrate amino acid displaces its lysyl residue (α-amino group), P-5-P transfers the aldimine linkage from the ε-amino group to the α-amino group. 

## 4. Transmethylation vs. Transsulfuration

### 4.1. Transmethylation and the One-Carbon Metabolism

Methylation is an essential metabolic function that controls the addition of –CH3 groups to a variety of organic compounds in every cell of the body. Transmethylation represents metabolic reactions in which –CH3 groups are transferred from one compound to another, comprising both demethylation and remethylation reactions. The methionine cycle contains good examples of transmethylation reactions, in which methionine is demethylated to homocysteine, *S*-adenosylmethionine acts as a methyl donor, with a further possible remethylation to methionine with either 5-methyl tetrahydrofolate or betaine as the methyl donor.

During the methionine cycle, the –CH3 group of methionine, which contains a sulfur atom, is activated by adenosine triphosphate (ATP) through the addition of adenosine to its sulfur. This reaction, which is catalyzed by methionine adenosyltransferase (MAT), forms *S*-adenosylmethionine (SAM) [25], which is an important donor of –CH3 groups to nucleic acids, proteins, and neurotransmitters. 

Upon transfer of its –CH3 group, SAM is rapidly converted to S-adenosylhomocysteine (SAH) that, by removal of the adenosine molecule catalyzed by SAH hydrolase (SAHH), is immediately hydrolyzed to homocysteine [26]. Homocysteine can then follow two major pathways; transsulfuration (see next sub-item) or remethylation (Figure 1). Two remethylation pathways regenerate methionine; one is independent of cobalamin (Cbl) but depends on betaine as the one-carbon donor, and the other is Cbl-dependent and requires folate (5-methyltetrahydrofolate) as the one-carbon donor [27].

Folate has two carbon-carbon double bonds that yield dihydrofolate (DHF) and tetrahydrofolate (THF) after saturation of its first and second carbon, respectively, by hydrogen. Folates serve as donors of single carbons in their reduced (5-methyl-THF; CH3THF), intermediate (5,10-methylene-THF; CH2THF), and oxidized (10-formyl-THF; CHOTHF) states [28]. Although not clearly shown in the literature [11,29,30], B_6_ status may influence the flux –CH3 groups through remethylation because 5,10-methylene-THF is formed by the methylene group of the side-chain of serine (after its conversion into glycine) along with THF in a reaction catalyzed by the B_6_-dependent enzyme serine hydroxylmethyltransferase. Although THF and serine, respectively, are the most important metabolic carrier and source of one-carbon groups, they do not promote the most energetically favorable reactions. The interaction of methionine with ATP produces SAM that easily donates its –CH3 group; SAM is considered the most important one-carbon donor [3]. Folate and SAM transfer of single carbons, which are volatile and bind easily to other molecules, generates the one-carbon metabolism. The one-carbon metabolism creates interplay between amino acid and nucleotide metabolisms, playing a fundamental role in DNA synthesis, repair, and replication [3]. The one-carbon donor 5-methyl-THF is used to convert homocysteine into methionine. The overall reaction transforms 5-methyl-THF into THF while transferring a –CH3 group to homocysteine to form methionine [4].

Methionine synthase (MTR), a vitamin B_12_ (Cbl)-dependent enzyme, catalyzes the final step in the regeneration of methionine from homocysteine [7]. The complex Cbl(I)MTR binds the –CH3 group of 5-methyl-THF to form methylCbl(III)MTR, activating the enzyme. The activated –CH3 group is transferred from methylCbl(III)MTR (regenerating Cbl(I)MTR) to homocysteine synthesizing methionine, which is released from the enzyme [31]. Under folate and/or vitamin B_12_ deficiency, MTR reactions are severely impaired.

### 4.2. Transsulfuration and the GPX System

As described above, after its synthesis from SAH, homocysteine can follow the remethylation or the transsulfuration pathway (Figure 1). Transsulfuration is a metabolic pathway involving the conversion of homocysteine into cysteine through the intermediate metabolite cystathionine. Briefly, in a reaction catalyzed by cystathionine β-synthase (CBS; B_6_-dependent enzyme), a β-replacement of the thiol group of homocysteine by the acetyl or succinyl group of homoserine forms cystathionine [32]. By acting at the homocysteine junction, CBS represents a critical step, regulating both the maintenance of the methionine pool and the synthesis of cysteine. Therefore, this enzyme would be expected to be strictly regulated. In fact, CBS is allosterically regulated by SAM, in which low SAM concentrations direct homocysteine into remethylation, whereas at high SAM concentrations, transsulfuration is favored [33]. Following cystathionine formation, the B_6_-dependent enzyme cystathionine γ-lyase (CGL) cleaves the molecule by γ-elimination of its homocysteine portion. This reaction leaves an unstable amino acid that binds to water molecules to form cysteine, α-ketobutyrate, and ammonia [34]. By the action of gamma-glutamylcysteine synthetase (GCL), cysteine and glutamate synthesize gamma-glutamylcysteine, a rate-limiting step in GSH synthesis [35]. Further, glycine binds to the C-terminal of gamma-glutamylcysteine via the enzyme GSH synthetase to form GSH.

Therefore, the transsulfuration pathway is a straight connection between homocysteine and GSH, the major redox buffer in mammalian cells. Consequently, it is expected that enzymes related to this metabolism display sensitivity to redox changes. Indeed, studies on purified mammalian MTR and CBS have revealed the reciprocal sensitivity of these two major homocysteine-utilizing enzymes to oxidative conditions [5,36]. In mammals, CBS contains a heme cofactor that functions as a redox sensor, increasing CBS activity and consequently transsulfuration under oxidizing conditions [36]. In contrast, under these same conditions, remethylation is depressed because MTR activity is reduced, most likely due to the lability of the reactive cofactor intermediate Cbl(I) [5]. Additionally, the betaine-related enzyme BHMT was also shown to be inhibited by oxidizing agents [6]. Considering that mammals metabolize seleno-amino acids in the same way as their sulfur counterparts [37], recent studies by this laboratory on the effects of selenium (Se) sources and levels combined or not with B_6_ provided indirect support to this effect of ROS. Dalto et al. [38] reported that, under the oxidative stress of ovulation, the gene expression of GPX1, 3, and 4 in the livers (Table 1) and kidneys (Table 2) of Se-supplemented animals were higher than in the control Se-unsupplemented group, and animals supplemented with organic Se plus B_6_ had the highest gene expression of GPX1 and selenocysteine lyase (SCLY), indicating that the transsulfuration pathway was stimulated. In contrast, Dalto et al. [39] observed, under basal oxidative stress conditions, no differential expression for the same genes (Table 1 and Table 2).

## 5. The Role of B_6_

### Differential Effects on Organic and Mineral Se Metabolisms in Gilts

Selenium is an essential trace element derived from inorganic (MSe) or organic (OSe) sources. Both forms are involved in the activation of SeGPX. The metabolism of Se-methionine, the natural organic source present in food, is interchangeable with a sulfur-methionine metabolism [37], and, therefore, the influence of B_6_ on the one-carbon metabolism by the regulation between remethylation and transsulfuration is predictable. Although less evident, selenide, which is the metabolized form of selenite (commonly used dietary MSe) and a key intermediate for the utilization and/or excretion of both OSe and MSe [40,41], may represent another regulatory step influencing one-carbon metabolism. In this sense, two important B_6_-dependent reactions direct selenide to be incorporated into Se-enzymes in preference to its excretion through the use of one-carbon groups in methylation reactions. 

For Se-methionine, it is converted to Se-homocysteine through the action of SAM synthetase and SAHH, supplying the one-carbon system with –CH3 groups (Figure 1). As discussed above for sulfur-homocysteine, Se-homocysteine may be remethylated to Se-methionine or transsulfurated to Se-cystathionine, depending on the influence of SAM levels and ROS feedback on CBS. In this context, the SAM levels may promote equilibrium between remethylation and transsulfuration depending on dietary OSe levels, whereas the positive feedback of ROS on CBS (and the negative feedback of MTR) favors the transsulfuration pathway independently of dietary Se levels. Se-methionine may also be transaminated to methylselenol and then transformed to selenide via methyltransferases [42] or methylated to excretory forms. However, considering the importance of this amino acid in protein synthesis and the vital consequences of transmethylation, the partition of its pool to transamination and methylation may have a secondary relevance.

After SeCys synthesis from Se-cystathionine via Se-cystathionine gamma lyase (CGL; B_6_-dependent enzyme), this amino acid can be incorporated into proteins or metabolized by the B_6_-dependent enzyme SeCys lyase (SCLY) to alanine and selenide [43]. Considering that SCLY is substrate specific, this step is the landmark between sulfur and Se metabolisms and settles their fates in relation to the metabolism of glutathione. It has to be stated that the greater volume of sulfur, rather than SeCys, molecules available in the organism makes the impact of B_6_ important not only for the enzyme SeGPX but also for its substrate (glutathione) as well.

Dietary selenite is non-enzymatically reduced via thiol-dependent reactions to selenide [44]. This direct reduction of selenite, short-cutting the transsulfuration pathway and with no regulatory mechanism, allows the accumulation of toxic levels of selenide. To avoid toxicity, selenide from both OSe (synthesized from SeCys) and MSe may follow two pathways: synthesis of Se-proteins or methylation (excretion). By the action of Se-phosphate syntethase 2 (B_6_-dependent enzyme), selenite is converted into Se-phosphate [45]. This last molecule acts as a Se donor in the exchange of the phosphate moiety of serine-tRNA for Se, generating the SeCys-specific tRNA(Ser)Sec, in a B_6_-dependent reaction [46]. This special tRNA, which contains the SeCys-insertion sequence (SECIS) along with a SeCys-tRNA-specific elongation factor (eEFSEC) and a specific SECIS binding protein (SECISBP2), is essential for SeCys incorporation into Se-proteins [47]. Remaining selenide must be successively methylated using –CH3 groups from SAM to generate the monomethylated intermediate methylselenol and multimethylated excretory metabolites (dimethylselenide and trimethylselenonium) [48].

Although the metabolism of OSe also generates selenide, the balance between SeCys incorporation into protein and its degradation by the saturable enzyme SCLY prevents the accumulation of excessive selenide. It is known that methylation and demethylation reactions between selenide and methylselenol promote equilibrium between the two molecules [41,42,49]; however, considering that low levels of dietary selenite supplementation provoke toxicity, whereas dietary Se-methionine is tolerated up to extreme high levels [50], one can assume that the utilisation of dietary selenite will direct the metabolism to consume greater amounts of one-carbon groups for the methylation of selenide than dietary Se-methionine. In contrast, OSe metabolism may not only preserve –CH3 molecules through the controlled synthesis of selenide, but also acts generating these one-carbon groups via demethylation/remethylation reactions between Se-methionine and Se-homocysteine. In this context, the regulatory steps (SAM levels, ROS feedback on CBS and MTR, and controlled synthesis of selenide from SCLY) in this metabolic pathway may promote a balance between the synthesis of one-carbon molecules and Se-proteins with possible beneficial effects to the organism.

Recent studies on Se and B_6_ metabolisms by this laboratory, using the peri-estrus period as a model for oxidative stress in gilts [38,39,51], provided peculiar information about the control of this metabolic pathway. Although those studies did not specifically address the homeostasis of –CH3 groups, based on the above information this review makes some indirect inferences about the one-carbon metabolism from responses reported in relation to the transsulfuration pathway.

According to Dalto et al. [38,39], B_6_ supplementation (10 mg/kg) does not affect the deposition of blood or tissue Se on a long-term basis (3 to 5 estrus), as well as blood Se levels during the peri-estrus period (oxidative stress condition) for both MSe and OSe. For MSe it is not surprising because, independently of the physiological state, absorbed selenite is quickly transformed into selenide and then converted into functional Se-proteins or methylated instead of being actively stored [9]. Consequently, the impact of MSe on the pool of one-carbon molecules for the methylation of selenide would be mainly related to the level of selenite supplementation rather than to B_6_ availability.

For OSe supplemented animals however, the explanation is not so straightforward. Under basal oxidative stress conditions, the absence of positive feedback of ROS on CBS suggests that the remethylation of Se-homocysteine would be the preferential pathway, whereas the high SAM levels would stimulate CBS to transsulfurate Se-homocysteine. However, according to Bekaert et al. [52], in humans, high doses of daily supplementation with selenium yeast (a source of OSe) over six months did not affect the homocysteine concentrations in plasma. Also, considering that CBS is a B_6_-dependent enzyme, its activity would direct more Se to SeGPX synthesis under B_6_ supplementation. Davis [53] and Lima [54] reported that CBS is slightly affected by marginal vitamin B_6_ status, whereas CGL is more sensitive, but neither of them affected cysteine synthesis. Although under marginal B_6_ levels, these studies indicate that the B_6_ status may not be a determining factor of the transsulfuration pathway flux, which is possibly mainly controlled by ROS and SAM levels. In fact, Dalto et al. [39] reported no effect of B_6_ on either blood SeGPX activity or GPX and SCLY gene expression in gilts at 30-days of gestation (Table 1 and Table 2), a conditions shown to be of low oxidative stress in this species. These results suggest that under basal oxidative stress conditions, in animals supplemented with Se and B_6_, neither remethylation nor transsulfuration but Se deposition into proteins is the major route for this mineral. 

In contrast, as also described above, Dalto et al. [38] reported that, under the oxidative stress of the peri-estrus period (3-days after estrus), GPX’s and SCLY genes were highly expressed in OSe + B_6_ supplemented animals in the liver and kidneys, in agreement with the positive feedback of ROS on CBS, increasing the ratio of transsulfuration. Interestingly, however, these same authors did not find any B_6_ effect on SeGPX activity in the liver and kidneys (day 3 after estus) or whole blood (day − 4 to day + 3 of the peri-estrus period). In this regard, Lubos et al. [55] reported that GPX can be regulated by transcriptional, post-transcriptional, translational, or post-translational factors, and changes in gene transcription may not be reflected on GPX protein or its related enzymatic activity.

## 6. Embryo Metabolism

### 6.1. 5-Days Porcine Embryos

Using microarray technology to evaluate the global gene expression in 5-days porcine embryos, Dalto et al. [56] provided unique and interesting information on Se and B_6_ metabolisms and, consequently, on the distinct interplay between remethylation and transsulfuration during early porcine embryo development.

The first aspect to be considered in 5-days embryos is the transfer of Se from dam to embryos. Dalto et al. [56] showed that Se was undetectable in the uterine flushing, indicating that this is a negligible source of this mineral for the pre-implantation embryo. Nevertheless, embryos from OSe supplemented dams are richer in Se than those from MSe supplemented dams. It was hypothesized that the most probable source is the pre-ovulatory oocyte that might be affected by the systemic maternal blood Se concentration through follicular fluid, considering that OSe is known to be more common deposited in tissues than is MSe [39,51,57,58]. In fact, Dalto et al. [56] observed that maternal supplementation with OSe plus B_6_ stimulated 28.8 times more genes than MSe plus B_6_ (six and 173 genes for MSe and OSe, respectively). These findings suggest that embryos coming from OSe supplemented dams have greater Se-methionine reserves and, therefore, are more suitable to go through demethylation steps to Se-homocysteine than are embryos from MSe supplemented dams.

**Figure 2:**
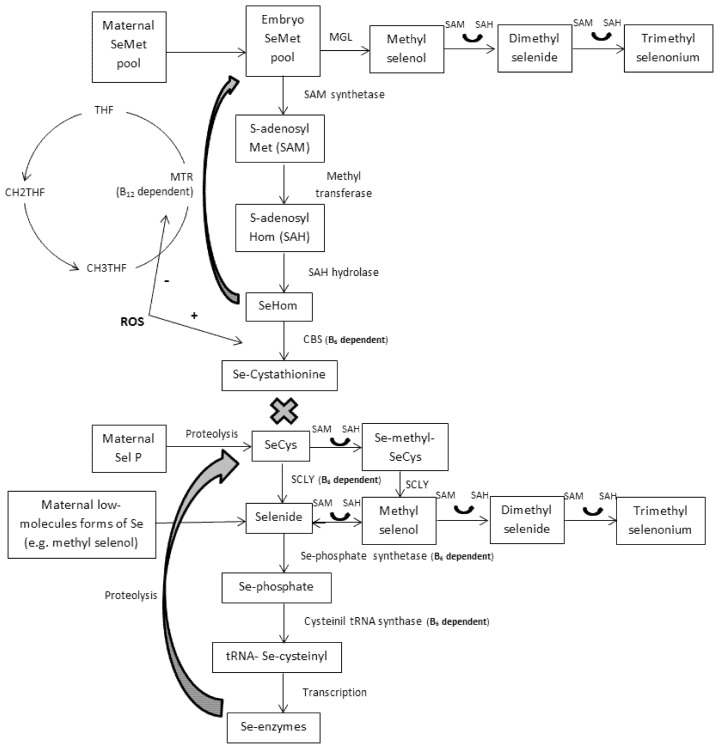
Embryo transmethylation and transsulfuration pathways of selenium (similar for sulfur counterparts) and possible maternal selenium transfer routes. Maternal selenomethionine (SeMet) is transferred to the embryo and may be methylated to methyl selenol by methionine γ lyase (MGL) or demethylated to selenohomocysteine (SeHom). SeHom may follow remethylation back to SeMet or transsulfuration to form selenocystathionine because the enzyme cystathionine γ lyase (CGL) that catalyzes the synthesis of selenocysteine (SeCys) from selenocystathionine is inactive in embryos. Pre-formed SeCys may be available after maternal selenoprotein P proteolysis in the placenta. Through the actions of selenocysteine lyase (SCLY), selenide is formed. Selenide may be methylated and excreted or phosphorylated and its selenium (Se) incorporated into tRNA Se-cysteinyl with further translation to Se-enzymes. THF = tetrahydrofolate; CH2THF = 5,10-methylene-THF; CH3THF = 5-methyl-THF; MTR = methionine synthase; CBS = cystathionine β synthase; ROS = reactive oxygen species.

A crucial aspect when studying the remethylation/transsulfuration pathway in embryos, fetuses, and newborns is the absence of CGL activity, in spite of its mRNA expression [10,14] (Figure 2). This relevant finding implies that, from conception to neonatal age, individuals are not able to convert Se-methionine into SeCys via the transsulfuration pathway. Therefore, Se-methionine would be directly incorporated into protein, methylated to methylselenol (with further methylation to excretory forms or demethylation to selenide), or demethylated to Se-homocysteine (generating the –CH3 donor SAM) and thereafter remethylated to Se-methionine via folate-dependent reactions, supplying the one-carbon metabolism. Dalto et al. [56] evaluated the expression of genes exclusively related to each source of Se (common genes between sources were excluded from the analysis) in 5-days embryos from gilts supplemented with OSe or MSe combined with B_6_ and did not find differentially expressed genes related to the demethylation or remethylation of Se-methionine, whereas several genes related to general elongation factors and biological processes related to translation and mitotic cell cycle were stimulated by OSe. Those authors concluded that, in 5-days porcine embryos, Se-methionine preferentially follows protein deposition. However, recent results from this laboratory (data not yet published), using the database generated by Dalto et al. [56], evaluated global gene expression in 5-days embryos (common genes between sources of Se were not excluded from the analysis), showing that OSe, but not MSe, may affect DNA methylation and epigenetic events in 5-days embryos through the higher generation of –CH3 groups (stimulation of methyltransferases and SAM carrier genes). Therefore, although Se-methionine may be preferentially incorporated into protein, the magnitude of the demethylation and remethylation reactions that compose the one-carbon metabolism cannot be disregarded. 

Although the transsulfuration pathway is not complete in embryos, many genes involved in Se-enzymes synthesis were found in Dalto et al. [56] suggesting that 5-days embryos are potentially capable of synthesizing these enzymes. If so, the most probable sources of Se to the embryo are SeCys, methylselenol, and/or Se-proteins (after proteolysis in the embryo) coming from the pre-ovulatory oocyte. Furthermore, SeGPX synthesis would possibly not be under the feedback control by ROS but limited by the B_6_-dependent enzyme SCLY, which controls the synthesis of selenide and, consequently, the consumption of one-carbon molecules for its methylation (Figure 2). Considering the importance of these one-carbon molecules for DNA processing and genetic stability, these routes of maternal Se transfer to early embryos may be considered a mechanism of defense to protect the pool of –CH3.

### 6.2. 30-Days Porcine Embryos

Similarly to 5-days embryos, the enzyme CGL is absent in 30-days embryos and, consequently, the above aspects related to remethylation/transsulfuration are expected to be analogous. However, at 30 days of gestation the presence of the placenta may change the dynamic of the interaction between these metabolic pathways.

One of the main features brought about by the presence of the placenta is the rise in the oxygen tension in the embryo, increasing the production of ROS. These levels are, in fact, higher in early gestation than thereafter [13], possibly because of the more immature embryonic antioxidant system. This suggests that, under oxidative stress conditions, the feedback of ROS stimulates CBS, directing Se-homocysteine to transsulfuration, whereas it would affect remethylation by reducing MTR activity. According to Kalhan [2], alterations to the metabolism of one-carbon may be the main cause of impaired fetal growth. Additionally, because the transsulfuration pathway is interrupted at Se-cystathionine, large amounts of this metabolite could possibly accumulate with negative effects in embryos from OSe supplemented dams. In this sense, under these conditions, B_6_ supplementation levels might be carefully considered due to its effect on CBS activity. Evidences of this negative effect of B_6_ on embryo development were observed in a study by Dalto et al. [39], in which maternal supplementation with 12.4 mg/kg of feed of B_6_, with either MSe or OSe at 0.6 mg/kg of feed, increased the within-litter Se content variation and the within-litter weight variation, compared to animals receiving 2.4 mg/kg of B_6_. Under similar experimental conditions and pig genetic lines, Fortier et al. [51] fed gilts with different sources of Se (MSe or OSe at 0.5 mg/kg of feed) but a fixed amount of B_6_ at 3 mg/kg and observed improved within-litter Se content variation, embryo weight and length, and protein and DNA content, with no detrimental effect on the within-litter weight variation, compared to the control diet at similar levels of B_6_ but 0.2 mg/kg of feed of Se.

As mentioned above, OSe increases body Se concentration more than MSe due to its deposition in proteins following the methionine metabolism, and B_6_ does not interfere with Se deposition in the tissues of adult individuals [38,39]. Also in 30-days porcine embryos, Fortier et al. [51] and Dalto et al. [39] showed that, independently of B_6_ status, gilts supplemented with OSe produced embryos with higher Se concentrations than gilts supplemented with MSe. Considering that the transsulfuration pathways is not complete at this stage of development, this higher load of Se-methionine in OSe embryos is not utilized in the synthesis of selenoenzymes but rather will be deposited in proteins and/or transmethylated, producing one-carbon groups. However, it has to be stated that even MSe supplemented gilts supply their embryos with OSe, not as Se-methionine but as SeCys, after selenoprotein P catabolism in the placenta [12]. In spite of the low activity of SCLY in vivo [59], one can assume that the majority of SeCys would be metabolized through SCLY because its concentration in tissues is enough to metabolize all SeCys available and the K_M_ (Michaelis constant) value of the enzyme is greater than the tissue levels of the substrate. However, Fortier et al. [51] and Dalto et al. [39] reported no effect of maternal Se levels (control vs. Se-supplemented gilts) and sources (OSe vs. MSe) on SeGPX activity in 30-days porcine embryos, indicating that at this stage of development the enzyme flux is not primarily substrate driven. Therefore, it is reasonable to hypothesize that embryo SeCys, from the catabolism of selenoprotein P in the placenta, follows primarily protein deposition and secondarily catabolism by SCLY, which is not controlled by the feedback of ROS, for the synthesis of selenide and further selenoenzymes. This mechanism would protect the embryo from wasting one-carbon groups for the methylation and elimination of excess selenide.

## 7. Conclusions

Both sulfur and Se methionine are important metabolic suppliers of one-carbon molecules through transmethylation reactions. The equilibrium between transmethylation and transsulfuration pathways, majorly regulated by redox conditions on CBS and MTR, influences the flow of –CH3 molecules between the one-carbon and antioxidation metabolisms.

Vitamin B_6_ is important for the interplay between the synthesis (transmethylation) and the utilization (transsulfuration) of one-carbon groups by acting in most of their regulatory enzymes. However, its effects on the GPX system depend on other parameters such as the oxidative stress conditions and metabolic maturity. 

Finally, whereas MSe may act by wasting one-carbon molecules to methylate the excess of selenide produced, OSe not only preserves one-carbon groups (due to the control of SCLY) but also promotes equilibrium between transsulfuration and transmethylation via the control of B_6_-dependent CBS by SAM and ROS.

## Figures and Tables

**Table 1:** Real-time mRNA abundance of liver glutathione peroxidase (GPX) and selenocysteine-lyase (SCLY) genes in gilts three days after the fourth estrus and at day 30 of gestation, according to selenium and vitamin B_6_ treatments.

	Day 3 Post-Estrus ^a^					30 Days Gestation ^b^				
	CONT	MSe B_6_0 ^c^	MSe B_6_10 ^c^	OSe B_6_0 ^c^	OSe B_6_10 ^c^	CONT	MSe B_6_0 ^c^	MSe B_6_10 ^c^	OSe B_6_0 ^c^	OSe B_6_10 ^c^
GPX1	0.44	1.02	1.09	0.77	1.57	1.15	1.19	1.37	1.27	1.15
GPX3	0.43	1.02	1.22	0.67	1.17	0.77	1.03	0.87	0.96	0.93
GPX4	0.59	1.00	1.08	0.66	1.19	1.05	1.05	1.19	1.07	0.98
SCLY	0.72	0.82	0.84	0.89	1.45	0.82	0.92	0.84	0.71	0.82

Adapted from Dalto et al. [38,39]. Standard error means for day three post estrus and 30 days gestation respectively equal 0.06 and 0.17 for GPX1, 0.05 and 0.12 for GPX3, 0.05 and 0.10 for GPX4, and 0.07 and 0.12 for SCLY. ^a^ For all treatments, GPX1, GPX3, GPX4, and SCLY were higher expressed than in the control diet (*p* < 0.01); Among all treatments, OSeB_6_10 presented the highest gene expression for GPX1 and SCLY (*p* < 0. 01); ^b^ No statistical difference (*p* ≥ 0.22); ^c^ CONT = basal diet; MSe = inorganic selenium; OSe = organic selenium.

**Table 2:** Real-time mRNA abundance of kidney glutathione peroxidase (GPX) and selenocysteine-lyase (SCLY) genes in gilts three days after the fourth estrus and at day 30 of gestation, according to selenium and vitamin B_6_ treatments.

	Day 3 Post-Estrus ^a^					30 Days Gestation ^b^				
	CONT ^c^	MSe B_6_0 ^c^	MSe B_6_10 ^c^	OSe B_6_0 ^c^	OSe B_6_10 ^c^	CONT ^c^	MSe B_6_0 ^c^	MSe B_6_10 ^c^	OSe B_6_0 ^c^	OSe B_6_10 ^c^
GPX1	0.72	1.16	1.25	1.19	1.83	0.97	1.11	0.95	1.03	1.14
GPX3	0.73	1.14	1.33	1.41	1.75	1.09	1.17	1.09	1.11	1.03
GPX4	1.06	1.12	1.18	1.18	1.47	1.10	1.22	1.12	1.18	1.17
SCLY	1.20	1.18	1.20	1.48	2.05	1.28	1.15	1.17	1.07	1.25

Adapted from Dalto et al. [38,39]. Standard error means for day three post estrus and 30 days gestation respectively equal 0.14 and 0.10 for GPX1, 0.10 and 0.09 for GPX3, 0.14 and 0.07 for GPX4, and 0.15 and 0.14 for SCLY. ^a^ For all treatments, GPX1 and GPX3 were higher (*p* < 0.01) expressed than control diet (*p* < 0.01); Among all treatments, OSeB_6_10 presented the highest gene expression for GPX1 and SCLY (*p* < 0.05); Only for GPX3, OSe was higher than MSe (*p* < 0.01) and B_6_10 was higher than B_6_0 (*p* < 0.01); ^b^ No statistical difference (*p* ≥ 0.18); ^c^ CONT = basal diet; MSe = inorganic selenium; OSe = organic selenium.
